# Divergent Roles of VEGF and TNF-α in Functional Impairment Among Patients with Carpal Tunnel Syndrome

**DOI:** 10.3390/ijms27114975

**Published:** 2026-05-30

**Authors:** Ireneusz Walaszek, Daria Schneider-Matyka, Mateusz Bosiacki, Szymon Grochans, Kaja Giżewska-Kacprzak, Elżbieta Grochans, Mariusz Panczyk, Anna Maria Cybulska, Patrycja Kapczuk, Kamila Rachubińska

**Affiliations:** 1 Department of Nursing, Faculty of Health Sciences, Pomeranian Medical University in Szczecin, 71-210 Szczecin, Poland; ireneusz.walaszek@pum.edu.pl (I.W.); elzbieta.grochans@pum.edu.pl (E.G.); anna.cybulska@pum.edu.pl (A.M.C.); kamila.rachubinska@pum.edu.pl (K.R.); 2 Department of Biochemistry, Faculty of Medicine, Pomeranian Medical University in Szczecin, 71-210 Szczecin, Poland; mateusz.bosiacki@pum.edu.pl (M.B.); patrycja.kapczuk@pum.edu.pl (P.K.); 3 Department of Pediatric and Oncological Surgery, Urology and Hand Surgery, Faculty of Medicine, Pomeranian Medical University in Szczecin, 71-252 Szczecin, Poland; szymon.grochans@pum.edu.pl (S.G.); kaja.gizewska.kacprzak@pum.edu.pl (K.G.-K.); 4 Department of Education and Research in Health Sciences, Faculty of Health Sciences, Medical University of Warsaw, 02-091 Warsaw, Poland; mariusz.panczyk@wum.edu.pl

**Keywords:** carpal tunnel syndrome, cytokines, VEGF, TNF-α, DASH questionnaire

## Abstract

Upper limb disorders, including carpal tunnel syndrome (CTS), are a common cause of pain, functional limitations, and reduced quality of life. Increasing attention has been directed toward the role of inflammatory and angiogenic mediators in the pathophysiology of CTS and their potential relationship with patient-reported functional outcomes. The aim of this study was to evaluate selected cytokines as potential markers of upper limb disability assessed using the Disabilities of the Arm, Shoulder and Hand (DASH) questionnaire. This cross-sectional study included 55 patients with idiopathic CTS referred for surgical treatment. Functional impairment was assessed using the DASH questionnaire, and concentrations of selected inflammatory and angiogenic mediators—*IL-4*, *IL-6*, *MCP-1*, *TNF-α*, *VEGF*, and *fraktalkine*—were measured in synovial tissue samples; these markers were selected based on their established roles in inflammation, angiogenesis, and nociceptive modulation relevant to CTS pathophysiology. VEGF was identified as a significant predictor of DASH category (*p* = 0.032), with higher concentrations associated with a lower likelihood of severe upper limb dysfunction. In contrast, higher TNF-α levels were associated with an increased risk of severe disability (*p* = 0.031). Other cytokines did not demonstrate significant associations with functional impairment. These findings suggest that selected inflammatory and angiogenic mediators were associated with the severity of functional disability in CTS and warrant investigation in prospective longitudinal studies to clarify their potential role alongside patient-reported outcome measures.

## 1. Introduction

Upper limb disorders are among the most common musculoskeletal problems, limiting professional and daily activities and reducing patients’ quality of life. Carpal tunnel syndrome (CTS) is the most common entrapment neuropathy and a significant source of upper limb disability, affecting occupational performance, daily activities and quality of life. In clinical practice and scientific research, patient-reported outcome measures (PROMs) are the standard for assessing the severity of symptoms and functional limitations, complementing objective performance tests and imaging. One of the most widely used tools is the Disabilities of the Arm, Shoulder and Hand (DASH) questionnaire, designed as a concise, self-report measure of symptoms and physical functioning in upper limb disorders of various aetiologies. At the same time, there is growing interest in biological markers of pathological processes accompanying upper limb injuries and overuse [1,2].

The pathophysiology of CTS involves complex mechanical mechanisms and biological mechanisms, with an inflammatory and microcirculatory component within the nerve and its surroundings [3]. In this context, particular importance is attached to cytokine mediators of inflammation (e.g., *TNF-α*, *IL-6*), which can modulate oedema, vascular permeability, nerve conduction and pain perception, and thus translate into functional outcomes measured by PROMs [4,5,6]. Data from populations exposed to chronic upper limb overload (computer operators) indicate that low-grade inflammation—with elevated *TNF-α* and *IL-6* concentrations—co-occurs with poorer hand function as assessed by DASH. Correlations between cytokine profiles and disability are also observed in neurological diseases, where elevated *IL-6/TNF-α* correlate with poorer functional outcomes, which reinforces the biological plausibility of such a relationship in CTS as well [7].

Many overload and degenerative-inflammatory conditions involve activation of the inflammatory axis and tissue remodelling. In this context, particular attention should be paid to pro-inflammatory cytokines and angiogenic factors, which may reflect the activity of the disease process and be associated with the severity of disability. Tumour necrosis factor alpha (*TNF-α*) is a key mediator of the inflammatory response, modulating pain, swelling, matrix degradation and nociceptive response. Vascular endothelial growth factor (*VEGF*) is responsible for angiogenesis and increased vascular permeability; its expression increases under conditions of ischaemia, microtrauma and tissue healing. Both factors—*TNF-α* and *VEGF*—represent exploratory biological markers of local tissue changes potentially associated with upper limb dysfunction in CTS, warranting evaluation in clinical cohorts [8].

Available studies have shown that in populations exposed to chronic upper limb overload, there is an association between inflammation (including elevated levels of TNF-α, IL-6 and C-reactive protein) and poorer functional outcomes as assessed by the DASH questionnaire [9,10]. Both conservative treatment and surgical treatment lead to improved PROMs of hand function [11,12,13]. However, there is still a lack of studies that simultaneously analyse changes in cytokine concentrations and the dynamics of functional impairment assessed using DASH or QuickDASH in homogeneous groups of patients with CTS.

The location of inflammatory biomarkers is crucial for their interpretation. Most of the studies conducted to date on cytokines in carpal tunnel syndrome have been based on measurements in peripheral blood, which reflect systemic inflammatory activity but do not always accurately reflect the local processes occurring within the carpal tunnel. Meanwhile, the pathophysiology of CTS is largely localised, involving inflammatory, microangiopathic and fibrotic changes within the tendon sheaths and perineural structures, including the synovium surrounding the median nerve. Cytokine measurements directly in the synovial tissue (synovium) may therefore provide more sensitive and biologically relevant information about the local inflammatory environment and its potential impact on nerve function and disability severity. In this study, cytokine concentrations (*IL-4*, *IL-6*, *MCP-1*, *TNF-α*, *VEGF* and *fractalkins*) were measured in synovial material, allowing for the assessment of the direct relationship between the local immune response and the degree of functional disability of the upper limb as assessed by the DASH scale [14,15,16].

Few studies have analysed the relationship between markers of angiogenesis and inflammation—in particular *VEGF* and *TNF-α*—and the level of upper limb disability in patients with CTS. Previous studies have focused predominantly on IL-6 and CRP, less often linking biomarkers directly with DASH scores or functional disability categories. Similarly, studies in adjacent musculoskeletal and neuropathic conditions have explored cytokine–disability associations, yet none have examined locally measured (synovial tissue) cytokine concentrations in relation to DASH-categorised functional disability in a surgical CTS cohort specifically. The aim of this study is therefore to evaluate the association between *VEGF*, *TNF-α*, and other selected inflammatory mediators measured in synovial tissue and upper limb dysfunction severity in patients with CTS, as an exploratory step towards integrating biomarkers with patient-reported outcome measures [11,12,13,14,15,16].

## 2. Results

### 2.1. Study Sample and Descriptive Statistics

The study included 55 participants, with a predominance of women (80.0%). The mean age was 58.96 ± 12.38 years, with a median of 58 years (IQR = 14). Regarding educational level, the largest group consisted of participants with secondary education (50.9%), followed by those with vocational education (27.3%). Most participants were married (72.7%), and resided in rural areas (32.7%) (Table 1 and Table 2).

The mean duration of symptoms from their onset was 3.96 ± 2.64 years. All participants presented with the cardinal symptom of CTS, supporting the validity of the study sample. The remaining symptom frequencies (numbness 47%, hand muscle weakness 36%, burning sensation 23%) are retained as they reflect the clinical heterogeneity relevant to functional disability interpretation (Table 3, Table 4 and Table 5).

The mean DASH score was 55.37 ± 14.14 points, indicating moderate-to-severe impairment of upper limb function. For regression analysis, DASH scores were categorised as follows: mild-to-moderate dysfunction (DASH ≤ 40, n = 12), severe dysfunction (DASH 41–70, n = 29), and severe disability (DASH > 70, n = 14). These thresholds are consistent with those applied in comparable CTS studies and reflect clinically meaningful gradations of upper limb disability.

### 2.2. Distributional Properties and Data Transformation

Prior to regression analysis, normality of cytokine concentrations was assessed using the Shapiro–Wilk test. All six mediators exhibited significant deviations from normality (all *p* < 0.05). Consequently, concentrations were subjected to Box–Cox transformation to stabilise variance and approximate normality, as described in the Section 4. Transformed values were subsequently used in all inferential analyses.

### 2.3. Multinomial Logistic Regression: Omnibus Tests

To assess the independent contribution of each cytokine to the prediction of DASH category, omnibus likelihood ratio χ^2^ tests were performed within the multinomial regression framework (Table 6).

Only VEGF was significantly associated with the level of functional disability (χ^2^(2) = 6.91, *p* = 0.032, η^2^ = 0.083). The effect size (η^2^ = 0.083) is of moderate magnitude by conventional statistical benchmarks (small ~0.01, medium ~0.06, large ~0.14), but its clinical relevance cannot be established from this exploratory, cross-sectional study and should not be interpreted as indicative of clinical significance. TNF-α showed a borderline result (χ^2^(2) = 5.76, *p* = 0.056, η^2^ = 0.069) that should be treated as hypothesis-generating only and does not constitute evidence of a true association. A similar non-significant trend was observed for *fractalkine* (χ^2^(2) = 5.56, *p* = 0.062, η^2^ = 0.067). *IL-4*, *IL-6*, and *MCP-1* did not show significant associations (*p* > 0.10). Critically, the overall model did not reach statistical significance (χ^2^(12) = 16.4, *p* = 0.175, R^2^ = 0.196), indicating that the cytokine panel explains only 19.6% of variability in functional disability; the majority of variability remains unexplained. In light of the non-significant overall model fit, all findings from individual predictors must be treated as exploratory.

It should be emphasised that statistical significance and effect magnitude, as reported here, do not equate to clinical relevance. The η^2^ values observed for *VEGF* (0.083) and *TNF-α* (0.069) fall within the moderate range by conventional benchmarks, yet the overall model explains less than 20% of variability in functional disability (R^2^ = 0.196), the sample is small (n = 55), and the design is cross-sectional. The clinical significance of these associations—including whether cytokine concentrations at the levels observed would translate into meaningful differences in patient outcomes—cannot be determined from the present data and requires investigation in prospective studies with larger cohorts and clinically anchored outcome thresholds.

### 2.4. Pairwise Contrast Analysis

**Figure 1:**
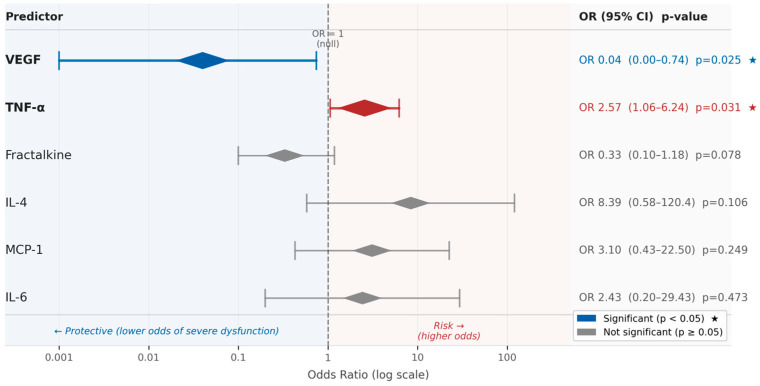
Forest plot: Odds Ratio for cytokine predictors of severe vs. mild dysfunction.

To assess directional effects, pairwise logistic contrasts were derived from the multinomial model for the comparison of “severe vs. mild dysfunction” and “severe disability vs. mild dysfunction” (Table 7, Figure 1).

In the analysis of specific contrasts, the comparison of ‘severe dysfunction vs. mild-to-moderate dysfunction’ showed that *VEGF* (OR = 0.04, 95% CI: 0.00–0.74, *p* = 0.025) and *TNF-α* (OR = 2.57, 95% CI: 1.06–6.24, *p* = 0.031) were statistically significant, whereas fractalkine did not reach statistical significance (*p* = 0.078). In the comparison of ‘severe disability vs. mild-to-moderate dysfunction’, none of the markers reached statistical significance, with non-significant trends for *VEGF* (*p* = 0.089) and *MCP-1* (*p* = 0.121) (Table 7). Given the exploratory nature of the analysis and the absence of multiple-comparison correction, all results should be treated as preliminary. The confidence intervals for several predictors are very wide, reflecting poor precision of estimates, rendering their point estimates unreliable and precluding meaningful interpretation. This instability is attributable to the small sample size and likely overfitting of the multinomial model; these results must therefore be disregarded for inferential purposes.

The reference category was “mild to moderate dysfunction”.

### 2.5. Model Fit and Adequacy

The overall model fit did not reach statistical significance (χ^2^(12) = 16.4, *p* = 0.175), indicating that the cytokine panel as a whole does not constitute a statistically adequate predictor of DASH category in this sample. The coefficient of determination R^2^ = 0.196 indicates that the model explains approximately 19.6% of the variability in functional disability—the large majority of the variability remains unexplained by the measured cytokines. Associations reported for individual predictors (*VEGF*, *TNF-α*) are exploratory observations that require replication in larger, adequately powered cohorts before any substantive conclusions can be drawn.

### 2.6. Exploratory and Sensitivity Power Analyses

Supplementary correlation analyses showed moderate-to-strong positive correlations among several cytokines, supporting the presence of a shared cytokine profile. However, continuous DASH scores were not significantly correlated with any individual cytokine in either Pearson or Spearman analyses after FDR correction (Supplementary File S1).

PCA supported a one-component solution for the cytokine panel. The data were suitable for PCA, with KMO = 0.802 and Bartlett’s test of sphericity χ^2^(15) = 140.175, *p* < 0.001. The retained component explained 57.33% of the total variance, and all cytokines loaded positively on this component. The component was interpreted as an overall inflammatory/angiogenic cytokine profile. However, the PCA-derived component score was not significantly associated with DASH scores, either in Pearson analysis, r = 0.020, 95% CI [−0.252, 0.288], *p* = 0.889, or Spearman analysis, ρ = −0.001, *p* = 0.995 (Supplementary File S2).

Sensitivity power analysis indicated that, for an effective sample size of N = 53–55, two-sided α = 0.05 and 80% power, the study was able to detect only moderate correlations, corresponding to a minimum detectable absolute correlation coefficient of approximately |r| = 0.365–0.371 (Supplementary File S2).

## 3. Discussion

Carpal tunnel syndrome is the most common type of compressive neuropathy of the median peripheral nerve and is characterised by a mixture of mechanical, vascular and biochemical mechanisms—including increased intracanal pressure, microcirculation disorders, tendon sheath oedema and chronic tissue remodelling within the carpal tunnel [17]. Increasing attention is also being paid to the role of inflammatory, angiogenic and neuroimmunological processes in the pathogenesis of CTS, including the activation of cytokines and growth factors that affect nerve and perineural tissue function [18]. Elucidating the relationship between cytokine profile and upper limb dysfunction severity, as assessed here using the DASH scale, may open up new prognostic and therapeutic opportunities in the treatment of CTS.

This study showed that among the analysed inflammatory and angiogenic markers, only the concentration of vascular endothelial growth factor (VEGF) was significantly associated with functional upper limb disability. These observations must be interpreted cautiously: the overall model did not reach statistical significance (*p* = 0.175), R^2^ = 0.196 indicates limited explanatory power, and all findings should be regarded as exploratory only. In the detailed contrast analysis, higher VEGF concentration was inversely associated with severe dysfunction in the ‘severe vs. mild-to-moderate’ comparison, suggesting a potential neuroprotective role, though this interpretation remains exploratory given the cross-sectional design. In turn, the level of TNF-α showed a significant positive association in the same contrast category. The other markers—IL-4, IL-6 and MCP-1—did not show significant associations with the DASH score.

### 3.1. VEGF

The inverse association between VEGF concentration and severe upper limb dysfunction is of interest and warrants careful interpretation. VEGF is commonly associated with angiogenesis, increased vascular permeability and nerve tissue regeneration—stimulating endothelial cell proliferation, improving tissue perfusion and supporting the survival of neurons and Schwann cells [19]. In the context of CTS, increased VEGF expression may represent a compensatory repair mechanism in response to median nerve ischaemia and chronic compression [20]. A study by Hirata et al. [19] showed increased VEGF expression in the tendon sheath in CTS, particularly in the transition phase between oedema and fibrosis. However, the possibility of reverse causation must be acknowledged: patients with less severe disability may retain greater capacity for compensatory VEGF upregulation, rather than VEGF directly causing lower disability. Furthermore, VEGF concentrations in this study were measured in synovial tissue at a single time point; longitudinal data would be needed to establish the direction of this association. In the detailed contrast analysis, higher VEGF concentration was inversely associated with severe dysfunction in the ‘severe vs. mild-to-moderate’ comparison. This inverse association may reflect a compensatory neuroprotective response to median nerve ischaemia in less severely affected patients; however, the cross-sectional design does not permit conclusions about biological protective action, causal direction, or temporal precedence.

### 3.2. TNF-α

The association between higher TNF-α concentrations and severe functional dysfunction is consistent with the known pro-inflammatory role of this cytokine. TNF-α is a key mediator of inflammation, demonstrated in models of peripheral nerve injury as a factor activating immune cells and provoking inflammation within the nerve [21]. In a study by Arshad et al. [1], plasma TNF-α and IL-6 levels were elevated in patients with CTS and correlated with the severity of nerve conduction changes, suggesting that TNF-α may reflect the active phase of nerve degeneration. However, given the cross-sectional design of the present study, this association cannot be interpreted as causal or prognostic; no directionality or temporal precedence can be established. The term ‘risk factor’ is therefore used here in a purely associational, non-causal sense.

These findings are not universally consistent with prior literature; Baričić et al. [22] reported no significant difference in TNF-α between CTS patients and controls measured in serum—a discrepancy likely reflecting the distinction between systemic and local (synovial tissue) inflammatory activity.

### 3.3. Fractalkine

The result for *fractalkine* (*CX3CL1*) showed only a statistical trend (*p* = 0.062) that did not meet the significance threshold; accordingly, this finding should not be over-interpreted. Fractalkine has a dual role—as a pro-inflammatory and neuroprotective factor—involved in neuron-microglia communication and the immune response in nervous tissues [23]. Notably, the *fractalkine/CX3CR1* system has also been linked to pancreatic β-cell function and insulin signalling, suggesting a potential connection with the metabolic comorbidities frequently observed in CTS (e.g., diabetes mellitus). This remains speculative and should be examined in adequately powered future studies.

### 3.4. Il-4, Il-6 and Mcp-1

The lack of significant associations between *IL-4*, *IL-6* and *MCP-1* levels and disability categories may indicate that these cytokines have limited value in the functional differentiation of upper limb dysfunction in patients with CTS within this cohort. IL-6, although widely recognised as an indicator of inflammatory activity, also has regenerative properties and its role in compressive neuropathies is unclear. In a study by Takasu et al. [24], elevated IL-6 concentrations were found in patients with CTS, while no differences in TNF-α were observed, which may suggest that these changes are time-dependent. The results of the study by Baričić et al. [22] showed no significant difference in *TNF-α* and *IL-1β* concentrations between CTS patients and a control group measured in serum. Importantly, the present study measured cytokine concentrations directly in synovial tissue, not in serum; local tissue concentrations reflect the intra-canal inflammatory environment more directly than systemic measurements. It is therefore possible that IL-4, IL-6 and *MCP-1* play a role in earlier stages of CTS pathogenesis, but their local tissue concentrations may not adequately discriminate between functional disability categories at the stage examined here.

The null findings for these cytokines are not fully consistent with prior reports; however, direct comparisons are limited by differences in biological compartment, disease stage, and outcome measures across studies. The cytokine–disability literature in CTS remains heterogeneous and the present findings should not be interpreted as definitive evidence of the absence of association.

### 3.5. Limitations

The advantage of this study is the measurement of cytokine concentrations directly in synovial tissue rather than in peripheral blood, which allows assessment of the local inflammatory environment within the carpal tunnel—a methodological strength given the localised pathophysiology of CTS. In addition, the use of Box–Cox transformation and multinomial logistic regression allowed for adequate consideration of functional categories and pairwise contrast comparisons. Nevertheless, the cross-sectional design precludes conclusions about the causal direction of the observed associations between biomarker concentrations and functional disability.

Nevertheless, a number of limitations should be emphasised. Firstly, the study sample is of moderate size (n = 55), which limits statistical power and may yield unstable regression estimates, as reflected in the extremely wide confidence intervals for some predictors (e.g., IL-6). Secondly, and critically, the overall regression model did not reach statistical significance (χ^2^(12) = 16.4, *p* = 0.175) and R^2^ = 0.196 indicates limited explanatory ability—the cytokine panel accounts for less than 20% of variability in functional disability. Conclusions drawn from individual predictors within a non-significant overall model must therefore be treated as strictly exploratory. The additional exploratory analyses provide important context for interpreting the regression findings. Although the cytokines formed a coherent one-component inflammatory/angiogenic profile, neither individual cytokine nor the PCA-derived component score was directly associated with continuous DASH scores. Therefore, the associations observed for TNF-α and VEGF in the multinomial regression model should be interpreted cautiously as conditional and category-dependent exploratory findings, rather than as evidence of simple linear or monotonic biomarker–disability relationships. Moreover, sensitivity power analysis indicated that the study was adequately powered only to detect moderate correlations, and smaller cytokine–DASH associations may have remained undetected.

Thirdly, the study did not include a healthy control group; all comparisons were performed internally between DASH-defined disability subgroups. Fourthly, no formal multiple-comparison correction was applied; borderline associations (TNF-α, fractalkine) must therefore be treated as hypothesis-generating only. Fifthly, data on potential confounders were not incorporated into the regression model, including CTS electrophysiological severity grade, hand dominance, BMI, and comorbidities (e.g., diabetes mellitus, rheumatoid arthritis)—all of which may independently influence both cytokine levels and DASH scores. Sixthly, the observed effect sizes (η^2^ = 0.083 for *VEGF*; η^2^ = 0.069 for *TNF-α*) are of moderate statistical magnitude by conventional benchmarks, but their clinical relevance cannot be established from this cross-sectional, exploratory study; moderate η^2^ does not imply clinical meaningfulness. Finally, the age of disease onset was not recorded separately; only symptom duration (mean 3.96 ± 2.64 years) was available, which limits disease-stage characterisation.

Fourthly, no formal multiple-comparison correction was applied to the primary multinomial regression and pairwise contrast analyses; therefore, borderline associations, particularly TNF-α and fractalkine, must be treated as hypothesis-generating only. In contrast, the supplementary correlation analyses were adjusted using the Benjamini–Hochberg FDR procedure.

From a preliminary and exploratory perspective, these findings raise the hypothesis that VEGF and TNF-α concentrations in synovial tissue may be relevant to the biological background of functional disability severity in CTS. However, given the modest explanatory power of the model (R^2^ = 0.196), the non-significant overall model fit, and the cross-sectional design, these results do not support clinical application at this stage. Replication in larger, prospective cohorts with appropriate correction for multiple comparisons and inclusion of potential confounders (e.g., diabetes, BMI, electrophysiological severity, hand dominance) is necessary before any translational conclusions can be drawn.

These findings are preliminary and should be interpreted with caution, given the exploratory nature of the study and the moderate sample size (n = 55); replication in larger, prospective cohorts is warranted.

## 4. Materials and Methods

This cross-sectional study was conducted among 55 patients diagnosed with unilateral or bilateral idiopathic CTS who were referred for surgical treatment to the Department of Orthopedics at the University Clinical Hospital No. 1 in Szczecin between 2024 and 2025. All participants signed an informed consent form prior to enrollment. Every effort was made to protect patient privacy and anonymity. The study was conducted in accordance with the current version of the Declaration of Helsinki. The study protocol was approved by the Bioethics Committee of the Pomeranian Medical University in Szczecin (KB-006/45/2023).

The study is part of a larger research project aimed at identifying biochemical, molecular, and psychosocial factors that affect the functioning and quality of life of patients with thumb carpometacarpal osteoarthritis and CTS.

Inclusion criteria for the study were: age over 18 years, body mass index (BMI) ≥ 18.5 kg/m^2^, a diagnosis of CTS with qualification for surgical treatment, and informed written consent provided before undergoing any study procedures.

Patients were excluded from the study if they met any of the following criteria: coexisting psychiatric disorders, including intellectual disability, organic brain dysfunction, or substance dependence (except for nicotine and caffeine); use of antipsychotic or antidepressant medications; participation in another clinical trial within 30 days prior to the initiation of or during the study; pregnancy or lactation; or any other reason for exclusion at the investigator’s discretion (e.g., insufficient compliance with the study procedures).

### 4.1. Study Design

The study is part of a larger research project—the materials and methods presented here refer exclusively to this part. It was conducted using a multi-stage design.

### 4.2. Stage One—Questionnaire Study

After obtaining approval from the Bioethics Committee to conduct the study, a trained interviewer personally distributed the previously prepared questionnaires to the participants. The respondents were informed about the purpose of the study and its anonymity, and were given the opportunity to ask questions and obtain comprehensive explanations. They then provided informed consent to participate.

This survey-based study employed a questionnaire technique. A standardized research instrument—the Disabilities of the Arm, Shoulder and Hand (DASH)—adapted for Polish conditions, was applied. The DASH is a 30-item self-report questionnaire assessing upper limb function. The questions concern activities of daily living, symptoms, and social functioning. Specifically, they address problems performing physically demanding activities with the upper limb (21 items), the severity of pain, numbness, weakness, and stiffness of the limb (5 items), as well as the impact of upper limb functional impairment on social activity, work, sleep, and self-perception. Responses are rated on a 5-point Likert scale, where 1 indicates no difficulty or symptoms and 5 indicates inability to perform the activity or extreme symptom severity. Scores for individual responses are summed to yield a total score ranging from 0 to 100, with higher scores indicating a greater degree of disability. Functional impairment was categorised into three groups based on DASH score: mild-to-moderate dysfunction (DASH < 40), severe dysfunction (DASH 40–70), and severe disability (DASH > 70). The DASH-based upper limb disability index is calculated according to the following formula: DASH Index = [(sum of item scores/number of completed items) − 1] × 25 [25].

A self-designed questionnaire was also used, comprising closed and semi-open questions aimed at collecting selected socio-demographic data, including age, education, marital status, place of residence, and employment status.

### 4.3. Stage Two—Tissue Collection During Surgical Procedure

The median nerve decompression procedure was performed through a surgical approach planned as a short, longitudinal skin incision of approximately 2–3 cm in length, located at the level of the wrist directly over the flexor retinaculum, distal to a line connecting the pisiform bone with the distal pole of the scaphoid. Soft tissues were dissected in layers. After exposing the operative field, the palmar surface of the flexor retinaculum, forming the roof of the carpal tunnel, was visualized.

Following longitudinal incision of the retinaculum, access to the contents of the carpal tunnel and complete visualization of the median nerve were achieved. Subsequently, a fragment of the flexor retinaculum, along with the adjacent synovial tissue of the flexor tendon sheaths, approximately 2 mm along the ulnar edge of the incised retinaculum, was excised. Tissue specimens obtained during the procedure, including fragments of the flexor retinaculum and synovial tissue, were preserved and submitted for diagnostic examination.

Once the tissue specimens were collected, the surgical wound was closed with interrupted sutures. A sterile dressing was applied to protect the operative site and provide moderate local compression. The arm tourniquet was then released, allowing controlled restoration of blood circulation to the limb.

### 4.4. Stage Three—Laboratory Analysis: Specimen Preparation and Study Procedure

Subsequently, the concentrations of selected inflammatory mediators, including cytokines (*IL-4*, *IL-6*, *MCP-1*, *TNF-α*) and angiogenic factors *(VEGF*, *fractalkine*), were determined in synovial tissue samples.

The concentrations of *IL-4*, *IL-6*, *MCP-1*, *TNF-α*, *VEGF*, and fractalkine were measured, with reference ranges established as follows: IL-4 between 31.25 and 2000 pg/mL, IL-6 between 4.688 and 300 pg/mL, TNF-α between 15.625 and 1000 pg/mL, *fractalkine* between 125 and 8000 pg/mL, VEGF between 31.25 and 2000 pg/mL and MCP-1 between 15.625 and 1000 pg/mL. Measurements were performed using commercially available ELISA kits (Wuhan Fine Biotech Co., Wuhan, China) according to the manufacturer’s instructions. The following kits were used: EH0199 for Human IL-4, EH0201 for Human IL-6, EH0302 for Human TNF-α, EH0141 for Human Fractalkine, EH0327 for Human VEGF, and EH0222 for Human MCP-1.

Assay sensitivities with intra- and inter-assay coefficients of variation (CVs) across all mediators were as follows: *IL-4*: 18.75 pg/mL, *CVs* of 5.4% and 5.7%, respectively; *IL-6*: 2.813 pg/mL, *CVs* of 4.66% and 4.64%, respectively; *MCP-1*: 9.375 pg/mL, *CVs* of 5.9% and 5.76%, respectively; *TNF-α:* 9.375 pg/mL, *CVs* of 5.99% and 5.85%, respectively; fractalkine: 75 pg/mL, *CVs* of 4.69% each; *VEGF*: 18.75 pg/mL, *CVs* of 5.64% and 5.94%, respectively.

### 4.5. Protein Concentration Measurement

Tissue fragments were homogenized using a pestle and a blade homogenizer (IKA T10 basic ULTRA-TURRAX®, IKA-Werke GmbH & Co. KG, Staufen, Germany) in 1.5 mL of PBS. The samples were then subjected to sonication (Sonics Vibra-Cell VCX 130, Sonics & Materials, Inc., Newtown, CT, USA; one 20 min cycle at 4 °C). The resulting extracts were centrifuged at 3000× *g* for 10 min at 4 °C. Protein concentration in the tissue was determined using the Micro BCA Protein Assay Kit (Thermo Scientific, Waltham, MA, USA). To obtain the supernatant, 2 µL of 50% NaOH was added to 23 µL of each tissue homogenate. The mixture was incubated in a heating block at 60 °C for 60 min. Subsequently, the first dilution was made (10 µL of supernatant + 90 µL of distilled water), and a 20 µL aliquot of the diluted sample was transferred to a well of a 96-well microplate. Next, 130 µL of distilled water and 150 µL of working reagent were added to each well in accordance with the kit protocol. The plate was incubated at 37 °C for 1.5 h. Absorbance was then measured at 562 nm using a microplate reader (BioTek Epoch 2 Microplate Spectrophotometer, Agilent Technologies, Santa Clara, CA, USA), and protein concentrations were calculated based on a bovine serum albumin (BSA) standard curve.

### 4.6. Statistical Analysis

All statistical analyses were performed using Jamovi software (version 2.6.44; The Jamovi Project). Descriptive statistics were calculated to characterize the study sample and the distribution of clinical and biochemical variables. Continuous variables were summarized using means and standard deviations (SD), and medians with interquartile ranges (IQR), whereas categorical variables were presented as absolute frequencies and percentages.

Prior to inferential analyses, the distributional properties of continuous predictors were examined, and due to deviations from normality, cytokine concentrations (*IL-4*, *IL-6*, *MCP-1*, *TNF-α*, *VEGF*, and *fractalkine*) were transformed using the Box–Cox transformation procedure to approximate normal distributions and stabilize variance [26].

The primary analytical objective was to assess the association between transformed cytokine levels and the degree of upper limb disability categorized according to the Disabilities of the Arm, Shoulder and Hand (DASH) score (mild to moderate dysfunction, severe dysfunction, severe disability). Given the nominal, three-level outcome variable without assuming proportional odds, multinomial logistic regression analysis was applied. The reference category was defined as “mild to moderate dysfunction,” and all independent variables were mean-centered prior to model estimation to reduce potential multicollinearity and facilitate interpretation of regression coefficients [27]. Omnibus likelihood ratio chi-square tests were used to evaluate the overall contribution of each predictor to the model.

Effect sizes for individual predictors in the multinomial logistic regression were estimated using η^2^ coefficients derived from the likelihood ratio χ^2^ statistics. The η^2^ index was calculated as η^2^ = χ^2^/(χ^2^ + N), where χ^2^ denotes the omnibus likelihood ratio test statistic for a given predictor and N represents the total sample size. This measure reflects the proportion of variability in the outcome attributable to a given predictor within the model. For descriptive interpretation, η^2^ values of approximately 0.01, 0.06, and 0.14 were considered indicative of small, medium, and large effects, respectively [28].

For specific pairwise contrasts between outcome categories, regression coefficients (β), standard errors (SE), odds ratios (OR), and corresponding 95% confidence intervals (CI) were calculated within the multinomial framework [29]. Model adequacy was evaluated using goodness-of-fit statistics, deviance, and information criteria, including the Akaike Information Criterion (AIC) and the Bayesian Information Criterion (BIC), as well as the coefficient of determination (R^2^) to assess explained variance. All statistical tests were two-tailed, and the level of statistical significance was set at α = 0.05; *p*-values below this threshold were considered statistically significant, whereas results approaching this threshold were interpreted with caution as indicating statistical trends.

Additional exploratory analyses were performed. Pairwise correlations were calculated between cytokine concentrations and continuous DASH scores, as well as among cytokines. Pearson correlations were computed using transformed cytokine concentrations, and Spearman’s rank correlations were used as a non-parametric sensitivity analysis. False discovery rate correction was applied using the Benjamini–Hochberg procedure.

Exploratory principal component analysis was conducted to determine whether the six measured cytokines could be summarized into a lower-dimensional cytokine profile. Sampling adequacy was assessed using the Kaiser–Meyer–Olkin measure, and factorability of the correlation matrix was evaluated using Bartlett’s test of sphericity. The number of components was determined using parallel analysis and scree plot inspection, with eigenvalues considered as supportive information. Component scores were subsequently correlated with continuous DASH scores.

Because the study was based on a fixed exploratory surgical cohort, a sensitivity power analysis was performed instead of observed post hoc power estimation. The analysis estimated the minimum detectable absolute correlation coefficient under two-sided α = 0.05 and 80% power.

## 5. Conclusions

The results of the present study indicate that patients with severe functional impairment of the upper limb in the course of carpal tunnel syndrome exhibit distinct alterations in their cytokine profile. In particular, severe dysfunction was associated with higher TNF-α levels and lower VEGF concentrations compared to patients with milder symptoms. This pattern may reflect, in cross-sectional terms, a predominance of pro-inflammatory and neurodegenerative mechanisms over angiogenic and regenerative processes in advanced stages of the disease.

The lack of significant differences for IL-4, IL-6, and MCP-1 suggests that not all components of the inflammatory response are directly linked to the severity of functional impairment, and their role may be secondary or dependent on specific stages of disease progression.

These findings do not challenge the primary importance of clinical history and physical examination in assessing the severity of dysfunction. Rather, they provide additional insight into the biological background of more advanced CTS. The cytokine profile may reflect the intensity of inflammatory and degenerative processes accompanying severe disease and could, in the future, serve as a complementary research tool for understanding pathomechanisms and identifying potential therapeutic targets. Further studies involving larger cohorts and longitudinal designs are warranted to confirm these associations and clarify their relevance in disease progression and treatment response. Given the cross-sectional design of this study, no causal inferences can be drawn from the observed associations between cytokine concentrations and functional disability severity.

## Figures and Tables

**Table 1:** Patient demographics and clinical characteristics (N = 55).

Parameter	Value	SD	Range
N (total)	55	—	—
Age (years)	58.0 (Me)	12.38	26–86
Disease duration (years)	3.0 (Me)	2.64	1–12
DASH score (points)	55.37 (M)	14.14	—

SD = standard deviation; IQR = interquartile range; DASH = Disabilities of the Arm, Shoulder and Hand.

**Table 2:** Socio-economic characteristics of study participants (N = 55).

Characteristic	n	%
Sex		
Female	44	80.0
Male	11	20.0
Education level		
Primary	4	7.3
Vocational	15	27.3
Secondary	28	50.9
Higher	8	14.5
Marital status		
Single	4	7.3
Married	40	72.7
Divorced	6	10.9
Widowed	5	9.1
Place of residence		
Village	18	32.7
Town < 10,000 inhabitants	9	16.4
Town 10,000–100,000 inhabitants	14	25.5
Town > 100,000 inhabitants	14	25.5
Employment status		
Employed	30	54.5
Retired	16	29.1
Unemployed	5	9.1
Disability pension	4	7.3

**Table 3:** Neurological symptoms reported by study participants (N = 55).

Symptom	Present (n)	%	Absent (n)	%
Tingling	55	100.0	0	0.0
Numbness	47	85.5	8	14.5
Hand muscle weakness	36	65.5	19	34.5
Burning sensation	23	41.8	32	58.2

**Table 4:** Disease duration—descriptive statistics (N = 55).

Median	Mean	SD	IQR	Min	Max
3.0	3.96	2.64	3.0	1	12

**Table 5:** Comorbidities (N = 55).

Comorbidity Status	n	%
With at least one comorbidity	36	65.5
No comorbidity	19	34.5

**Table 6:** Omnibus likelihood ratio χ^2^ tests from multinomial logistic regression for Box–Cox–transformed cytokine concentrations predicting DASH level.

Variable	χ^2^	*p*	η^2^
VEGF [pg/mg]_trans	6.91	0.032	0.0829
TNF-α [pg/mg]_trans	5.76	0.056	0.0691
Fractalkine [pg/mg]_trans	5.56	0.062	0.0667
IL-4 [pg/mg]_trans	4.57	0.102	0.0548
MCP-1 [pg/mg]_trans	2.82	0.244	0.0339
IL-6 [pg/mg]_trans	2.56	0.278	0.0307

χ^2^—chi-square test statistic; η^2^—effect size index derived from the omnibus test, expressing the proportion of variability attributable to the predictor in the context of the model; larger η^2^ values indicate stronger associations.

**Table 7:** Logistic regression parameters for transformed cytokine concentrations as predictors of the level of disability (DASH).

				95% Confidence Interval				
Comparison	Predictor	β	SE	Lower	Upper	OR	z	*p*
Severe vs. mild	Intercept	9.12	0.65	2.47	33.64	2.21	3.425	<0.001
	IL-4 [pg/mg]_trans	8.39	1.32	0.58	120.37	2.13	1.615	0.106
	IL-6 [pg/mg]_trans	2.43	1.23	0.20	29.43	0.89	0.718	0.473
	MCP-1 [pg/mg]_trans	3.10	0.98	0.43	22.50	1.13	1.154	0.249
	TNF-α [pg/mg]_trans	2.57	0.44	1.06	6.24	0.94	2.157	0.031
	VEGF [pg/mg]_trans	0.04	1.40	0.00	0.74	−3.13	−2.242	0.025
	Fractalkine [pg/mg]_trans	0.33	0.62	0.10	1.18	−1.09	−1.761	0.078
Severe vs. Mild	Intercept	1.02	0.84	0.19	5.59	0.02	0.024	0.981
	IL-4 [pg/mg]_trans	1.22	1.73	0.04	40.08	0.20	0.116	0.908
	IL-6 [pg/mg]_trans	25.72	2.20	0.30	2182.79	3.25	1.479	0.139
	MCP-1 [pg/mg]_trans	8.71	1.40	0.52	146.34	2.16	1.551	0.121
	TNF-α [pg/mg]_trans	1.88	0.56	0.60	5.85	0.63	1.122	0.262
	VEGF [pg/mg]_trans	0.05	1.78	0.00	1.78	−3.03	−1.698	0.089
	Fractalkine [pg/mg]_trans	0.41	0.76	0.09	1.88	−0.90	−1.191	0.234

Β—regression Coefficient; SE—standard Error; OR—odds Ratio.

## Data Availability

The data presented in this study are available on request from the corresponding author. The data are not publicly available due to ethical restrictions related to patient privacy and the terms of the study protocol approved by the Bioethics Committee of the Pomeranian Medical University in Szczecin (KB-006/45/2023).

## Supplementary Materials

The following supporting information can be downloaded at: https://www.mdpi.com/article/10.3390/ijms27114975/s1.

## Abbreviations

The following abbreviations are used in this manuscript:

CTS	The Carpal Tunnel Syndrome
DASH	The Disabilities of the Arm, Shoulder and Hand
PROMs	Patient-Reported Outcome Measures
TNF-α	Tumour Necrosis Factor Alpha
VEGF	Vascular Endothelial Growth Factor

## References

[1] Arshad M.S., Mattoo B., Alam I. (2024). Exploring pathogenic pathways in carpal tunnel syndrome: Sterile inflammation and oxidative stress. J. Basic Clin. Physiol. Pharmacol..

[2] Zafar R. (2021). Level of functional impairment using symptom severity and functional status scales in patients with carpal tunnel syndrome. Heal. J. Physiother. Rehabil. Sci..

[3] Yang C.F., Pu Y., Li L., Guo M.G., Feng Z.W. (2024). Inflammatory cytokines and carpal tunnel syndrome: A causal relationship revealed. Cytokine.

[4] Riondino S., La Farina F., Martini F., Guadagni F., Ferroni P. (2011). Functional impairment in video terminal operators is related to low-grade inflammation. Int. Arch. Occup. Environ. Health.

[5] Băcilă C., Vlădoiu M.-G., Valeanu M., Moga D., Pumnea P.-M. (2025). The role of IL-6 and TNF-α biomarkers in predicting disability outcomes in acute ischemic stroke patients. Life.

[6] Li J.-Y., Xue H.-R., Wang L., Zhang M.N., Zhang Y.Y. (2023). Relationship of immune cells with disability and cognitive impairment in NMOSD. Eur. Rev. Med. Pharmacol. Sci..

[7] Karimi N., AbedianKenari S., Darvari F. (2020). Serum levels of inflammatory cytokines in patients with idiopathic carpal tunnel syndrome. Int. J. Neurosci..

[8] Sandy-Hindmarch O., Bennett D., Wiberg A., Furniss D., Baskozos G., Schmid A. (2021). Systemic inflammatory markers in neuropathic pain, nerve injury and recovery. Pain.

[9] Shin Y., Yoon J., Kim Y., Kim J. (2018). Psychological status is associated with symptom severity in patients with carpal tunnel syndrome. J. Hand Surg..

[10] Daliri B.O., Azhari A., Khaki S., Hajebi Khaniki S., Moradi A. (2021). Which psychological and electrodiagnostic factors are associated with limb disability in patients with carpal tunnel syndrome?. Clin. Orthop. Relat. Res..

[11] Hamzeh H., Madi M., Alghwiri A.A., Hawamdeh Z. (2020). The long-term effect of neurodynamics vs exercise therapy on pain and function in CTS: Randomised parallel-group trial. J. Hand Ther..

[12] Mamipour H., Negahban H., Badiee Aval S., Zaferanieh M., Moradi A., Kachooei A. (2023). Physiotherapy plus acupuncture vs physiotherapy alone in CTS: Randomised clinical trial. J. Bodyw. Mov. Ther..

[13] Tadeusz Wielemborek P., Kapica-Topczewska K., Pogorzelski R., Bartoszuk A., Kułakowski R., Mirowska-Guzel D., Kułakowska A. (2022). Manual therapy improves symptom severity and disability in CTS. Neurol. Neurochir. Pol..

[14] Muñoz-Gómez E., Aguilar-Rodríguez M., Inglés M., Mollà-Casanova S., Sempere-Rubio N., Serra-Añó P. (2023). Effects of mirror therapy on pain, sensitivity and functionality in unilateral CTS: Randomised controlled trial. Disabil. Rehabil..

[15] Donati D., Goretti C., Tedeschi R., Boccolari P., Ricci V., Farì G., Tarallo L. (2024). Comparing endoscopic and conventional surgery techniques for CTS: Retrospective study. JPRAS Open.

[16] Guo T., Li C., Tian D., Gao R., Yu K., Sun N., Bai J. (2025). Device-assisted mini-incision versus conventional carpal tunnel release: Retrospective cohort of 109 cases. Ther. Clin. Risk Manag..

[17] Aboonq M.S. (2015). Pathophysiology of carpal tunnel syndrome. Neurosciences.

[18] Moalem-Taylor G., Baharuddin B., Bennett B., Krishnan A.V., Huynh W., Kiernan M.C., Lin C.S.-Y., Shulruf B., Keoshkerian E., Cameron B. (2017). Immune dysregulation in patients with carpal tunnel syndrome. Sci. Rep..

[19] Hirata H., Nagakura T., Tsujii M., Morita A., Fujisawa K., Uchida A. (2004). The relationship of VEGF and PGE2 expression to extracellular matrix remodelling of the tenosynovium in carpal tunnel syndrome. J. Pathol..

[20] Zimmerman M., Gottsäter A., Dahlin L.B. (2022). Carpal tunnel syndrome and diabetes—A comprehensive review. J. Clin. Med..

[21] Wang Y., Guo L., Yin X., McCarthy E.C., Cheng M.I., Hoang A.T., Chen H.C., Patel A.Y., Allard Trout D., Xu E. (2022). Pathogenic TNF-α drives peripheral nerve inflammation in an Aire-deficient model of autoimmunity. Proc. Natl. Acad. Sci. USA.

[22] Baričić M., Cvijanović Peloza O., Jerbić Radetić A.T., Šantić V., Omrčen H., Zoričić Cvek S. (2023). Serum levels of inflammatory and fibrotic cytokines in patients with carpal tunnel syndrome and hip osteoarthritis. Biomedicines.

[23] Lauro C., Catalano M., Di Paolo E., Chece G., De Costanzo I., Trettel F., Limatola C. (2015). Fractalkine/CX3CL1 engages different neuroprotective responses upon selective glutamate receptor overactivation. Front. Cell. Neurosci..

[24] Takasu S., Takatsu S., Kunitomo K., Kokumai Y. (1994). Serum hyaluronic acid and interleukin-6 as possible markers of carpal tunnel syndrome in chronic haemodialysis patients. Artif. Organs.

[25] Golicki D., Krzysiak M., Strzelczyk P. (2014). Translation and cultural adaptation of the Polish versions of the Disabilities of the Arm, Shoulder and Hand (DASH) and QuickDASH questionnaires. Ortop. Traumatol. Rehabil..

[26] Sakia R.M. (1992). The Box–Cox transformation technique: A review. J. R. Stat. Soc. Ser. D..

[27] Cohen J., Cohen P., West S.G., Aiken L.S. (2013). Applied Multiple Regression/Correlation Analysis for the Behavioral Sciences.

[28] Johnston J.E., Berry K.J., Mielke P.W. (2006). Measures of effect size for chi-squared and likelihood-ratio goodness-of-fit tests. Percept. Mot. Ski..

[29] Agresti A. (2013). Categorical Data Analysis.

