# Supplementary material for: Divergent Roles of VEGF and TNF-α in Functional Impairment Among Patients with Carpal Tunnel Syndrome
Source: Int J Mol Sci. 2026 May 30;27(11):4975. doi: 10.3390/ijms27114975 (PMC13257110; doi:10.3390/ijms27114975)
Supplement: Supplementary file 1 [file ijms-27-04975-s001.zip › Supplementary_File_1_Additional_Analyses.pdf]

Supplementary Table S1. Pairwise correlations between transformed cytokine concentrations and DASH score

Pearson correlations were calculated using transformed cytokine concentrations; Spearman correlations are presented as a rank-based sensitivity analysis. FDR-adjusted q-values were calculated using the Benjamini–Hochberg procedure separately for the Pearson and Spearman correlation families across 21 pairwise comparisons.

Variable 1	Variable 2	N	Pearson r	p	qFDR	Spearman ρ	p	qFDR
Fractalkine [pg/mg]_trans	IL-4 [pg/mg]_trans	54	0.545	<0.001	0.002	0.501	<0.001	0.002
Fractalkine [pg/mg]_trans	IL-6 [pg/mg]_trans	54	0.620	<0.001	0.002	0.599	<0.001	0.002
Fractalkine [pg/mg]_trans	MCP-1 [pg/mg]_trans	54	0.566	<0.001	0.002	0.550	<0.001	0.002
Fractalkine [pg/mg]_trans	TNF-α [pg/mg]_trans	54	0.551	<0.001	0.002	0.538	<0.001	0.002
Fractalkine [pg/mg]_trans	VEGF [pg/mg]_trans	53	0.457	<0.001	0.002	0.447	<0.001	0.002
Fractalkine [pg/mg]_trans	DASH	54	-0.021	0.878	0.878	-0.021	0.883	0.901
IL-4 [pg/mg]_trans	IL-6 [pg/mg]_trans	54	0.512	<0.001	0.002	0.493	<0.001	0.002
IL-4 [pg/mg]_trans	MCP-1 [pg/mg]_trans	54	0.676	<0.001	0.002	0.742	<0.001	0.002
IL-4 [pg/mg]_trans	TNF-α [pg/mg]_trans	54	0.366	0.007	0.011	0.332	0.014	0.023
IL-4 [pg/mg]_trans	VEGF [pg/mg]_trans	53	0.692	<0.001	0.002	0.722	<0.001	0.002
IL-4 [pg/mg]_trans	DASH	54	-0.096	0.488	0.603	-0.081	0.558	0.689
IL-6 [pg/mg]_trans	MCP-1 [pg/mg]_trans	54	0.502	<0.001	0.002	0.493	<0.001	0.002
IL-6 [pg/mg]_trans	TNF-α [pg/mg]_trans	54	0.453	<0.001	0.002	0.446	<0.001	0.002
IL-6 [pg/mg]_trans	VEGF [pg/mg]_trans	53	0.442	<0.001	0.002	0.482	<0.001	0.002
IL-6 [pg/mg]_trans	DASH	54	0.032	0.820	0.878	-0.029	0.836	0.901
MCP-1 [pg/mg]_trans	TNF-α [pg/mg]_trans	54	0.188	0.173	0.242	0.166	0.230	0.322
MCP-1 [pg/mg]_trans	VEGF [pg/mg]_trans	53	0.729	<0.001	0.002	0.742	<0.001	0.002
MCP-1 [pg/mg]_trans	DASH	54	0.024	0.862	0.878	0.017	0.901	0.901
TNF-α [pg/mg]_trans	VEGF [pg/mg]_trans	53	0.221	0.112	0.168	0.194	0.165	0.248
TNF-α [pg/mg]_trans	DASH	54	0.117	0.398	0.522	0.133	0.337	0.442
VEGF [pg/mg]_trans	DASH	53	-0.033	0.814	0.878	-0.066	0.640	0.747

Note. N was inferred from the degrees of freedom in the source matrix (N = df + 2), indicating pairwise complete observations. qFDR = Benjamini–Hochberg false discovery rate-adjusted p-value. For source p-values reported as <0.001, p = 0.001 was used for conservative FDR calculation; therefore, q-values for these rows are conservative. DASH = Disabilities of the Arm, Shoulder and Hand; MCP-1 = monocyte chemoattractant protein-1; TNF-α = tumor necrosis factor alpha; VEGF = vascular endothelial growth factor.
