# Supplementary material for: Divergent Roles of VEGF and TNF-α in Functional Impairment Among Patients with Carpal Tunnel Syndrome
Source: Int J Mol Sci. 2026 May 30;27(11):4975. doi: 10.3390/ijms27114975 (PMC13257110; doi:10.3390/ijms27114975)
Supplement: Supplementary file 1 [file ijms-27-04975-s001.zip › Supplementary_File_2_Additional_Analyses.pdf]

S2.1. Principal component analysis of cytokine concentrations

PCA was conducted on transformed cytokine concentrations to examine whether the six measured markers could be summarized into a lower-dimensional cytokine profile. Sampling adequacy was assessed using the Kaiser–Meyer–Olkin (KMO) measure, and factorability of the correlation matrix was evaluated using Bartlett's test of sphericity. The number of retained components was determined based on parallel analysis and scree plot inspection, with eigenvalues considered as supportive information.

Table S2.1. PCA assumption checks

Assumption check	Value	Interpretation
KMO, overall	0.802	Good sampling adequacy for PCA
Bartlett's test of sphericity	$\chi^2(15) = 140.175$, $p < .001$	Correlation matrix differs from an identity matrix

Note. KMO = Kaiser–Meyer–Olkin measure of sampling adequacy.

Table S2.2. Individual measures of sampling adequacy

Variable	MSA
Fractalkine	0.798
IL-4	0.855
IL-6	0.886
MCP-1	0.769
TNF-α	0.685
VEGF	0.793

Note. MSA = measure of sampling adequacy. All variables met acceptable sampling adequacy criteria.

Table S2.3. Initial eigenvalues and explained variance

Component	Eigenvalue	% of variance	Cumulative %
1	3.440	57.328	57.328
2	1.086	18.093	75.421
3	0.525	8.754	84.175
4	0.409	6.815	90.991
5	0.302	5.032	96.023
6	0.239	3.977	100.000

Note. Although the second component had an eigenvalue slightly above 1.0, parallel analysis and scree plot inspection supported a one-component solution. The retained component explained 57.33% of the total variance.

Table S2.4. Component loadings for the retained one-component PCA solution

Variable	Component 1 loading	Uniqueness
Fractalkine	0.791	0.374
IL-4	0.820	0.327
IL-6	0.746	0.444
MCP-1	0.800	0.360
TNF-α	0.546	0.702
VEGF	0.804	0.353

Note. All cytokines loaded positively on Component 1. Loadings ≥ |0.40| were considered salient. The retained component was interpreted as an overall inflammatory/angiogenic cytokine profile.

S2.2. Association between PCA Component 1 score and DASH

After extracting the one-component PCA solution, component scores were correlated with continuous DASH scores to evaluate whether the overall cytokine profile was associated with patient-reported upper limb disability.

Table S2.5. Correlation between PCA Component 1 score and DASH

Analysis	N	df	Effect estimate	95% CI	p-value
Pearson correlation	53	51	r = 0.020	−0.252 to 0.288	0.889
Spearman correlation	53	51	ρ = −0.001	N/A	0.995

Note. N was inferred from df + 2. The PCA-derived component score was not significantly associated with continuous DASH scores using either Pearson or Spearman correlation.

Although the cytokines formed a coherent one-component profile, this global cytokine component was not directly associated with DASH. This result is consistent with the absence of significant bivariate correlations between individual cytokines and DASH in Supplementary Table S1.

S2.3. Sensitivity power analysis

Because the study was based on a fixed exploratory surgical cohort, sensitivity power analysis was preferred over observed post-hoc power. The analysis estimated the minimum detectable absolute correlation coefficient under two-sided α = .05 and 80% power.

Table S2.6. Minimum detectable correlation coefficients

Effective sample size	Assumptions	Minimum detectable |r|	Interpretation
N = 55	Two-sided α = .05; power = 80%	0.365	Moderate association
N = 54	Two-sided α = .05; power = 80%	0.368	Moderate association
N = 53	Two-sided α = .05; power = 80%	0.371	Moderate association

Note. Values were calculated for a two-sided correlation test. Because some pairwise analyses used N = 53–54 due to pairwise complete observations, the minimum detectable effect was also reported for these effective sample sizes.

The study had adequate sensitivity to detect only moderate correlations. It was likely underpowered to identify small cytokine–DASH associations. Therefore, non-significant correlation results should be interpreted in the context of limited precision, whereas regression findings should be considered exploratory.
